# Combined Filtering Method for Offshore Oil and Gas Platform Point Cloud Data Based on KNN_PCF and Hy_WHF and Its Application in 3D Reconstruction

**DOI:** 10.3390/s24020615

**Published:** 2024-01-18

**Authors:** Chunqing Ran, Xiaobo Zhang, Hao Yu, Zhengyang Wang, Shengli Wang, Jichao Yang

**Affiliations:** 1 College of Ocean Science and Engineering, Shandong University of Science and Technology, Qingdao 266590, China; ranchunqing@sdust.edu.cn (C.R.); yuhao77@sdust.edu.cn (H.Y.); wangzhengyang@sdust.edu.cn (Z.W.); shlwang@sdust.edu.cn (S.W.); 2 College of Geodesy and Geomatics, Shandong University of Science and Technology, Qingdao 266590, China

**Keywords:** combinatorial filtering, point cloud data, offshore oil and gas platform, K-Nearest-Neighborhood point cloud filtering (KNN_PCF) algorithm, hybrid filtering with hyperbolic function weighting (Hy_WHF) algorithm, 3D reconstruction

## Abstract

With the increasing scale of deep-sea oil exploration and drilling platforms, the assessment, maintenance, and optimization of marine structures have become crucial. Traditional detection and manual measurement methods are inadequate for meeting these demands, but three-dimensional laser scanning technology offers a promising solution. However, the complexity of the marine environment, including waves and wind, often leads to problematic point cloud data characterized by noise points and redundancy. To address this challenge, this paper proposes a method that combines K-Nearest-Neighborhood filtering with a hyperbolic function-based weighted hybrid filtering. The experimental results demonstrate the exceptional performance of the algorithm in processing point cloud data from offshore oil and gas platforms. The method improves noise point filtering efficiency by approximately 11% and decreases the total error by 0.6 percentage points compared to existing technologies. Not only does this method accurately process anomalies in high-density areas—it also removes noise while preserving important details. Furthermore, the research method presented in this paper is particularly suited for processing large point cloud data in complex marine environments. It enhances data accuracy and optimizes the three-dimensional reconstruction of offshore oil and gas platforms, providing reliable dimensional information for land-based prefabrication of these platforms.

## 1. Introduction

The application of 3D laser scanning technology on offshore oil and gas platforms has garnered increasing attention from the industrial sector due to advancements in engineering technology [1]. By utilizing 3D point cloud data obtained from scanning, a more accurate representation of real objects and scene information can be achieved, yielding precise inputs for subsequent tasks such as structural analysis, fault prediction, and other related analyses [2]. Moreover, this technology establishes a robust data foundation for the “prefabrication on land, installation at sea” strategy [3]. However, the collection of data on offshore oil and gas platforms is challenged by the complex and adverse marine environment [4], characterized by water surface fluctuations, sea breezes, and changes in environmental lighting [5]. These environmental factors often result in the presence of isolated noise points, surface reflection noise points, and noise points in coefficient areas within the data [6]. Such noise significantly diminishes the effectiveness of the technology on offshore oil and gas platforms, emphasizing the critical importance of implementing effective filtering techniques [7].

In their early research, Orts-Escolano et al. [8] introduced a filtering method based on a voxel grid to effectively reduce the volume of point cloud data. However, Moorfield et al. [9] raised concerns about the possible loss of important information, particularly in handling intricate parts. To address this limitation, some filtering methods incorporated normal information into the filtering process to achieve more detailed manipulation of the point cloud data. Nonetheless, these approaches still encountered challenges when processing large-scene point cloud data and lacked the ability to emphasize local point cloud density. Subsequent studies introduced radius filtering [10] and statistical filtering [11] as alternative techniques. While these methods addressed the low computational efficiency in handling large scenes and were capable of removing most outlier noise points, they still had shortcomings. For instance, radius filtering exhibited inaccuracies in sparse areas, while statistical filtering struggled to eliminate densely-clustered noise points [12].

In order to effectively address the challenges presented by complex scenarios involving large-scale point cloud data exhibiting diverse noise types and uneven point cloud density distribution, researchers have started exploring the integration of multiple filtering strategies [13,14,15,16,17,18,19] to develop more stable and computationally efficient methods. However, it is important to note that the effectiveness of these combined approaches varies depending on the specific application scenario. In the case of offshore oil and gas platforms, which encompass structures such as pipelines and flanges, the data volumes are significant, and the data collection environments are complex [20]. These factors not only increase the complexity of processing the point cloud data but also have the potential to impact the accuracy of subsequent analysis and decision-making processes.

To address these challenges, this paper presents a novel filtering algorithm that combines the KNN_PCF approach [21] with the Hy_WHF algorithm scheme [22]. The KNN_PCF algorithm effectively discriminates between noise and the genuine structure of the platform by taking into account the relative positions and densities of data points. On the other hand, the Hy_WHF, offers a flexible means of weight distribution. This enables the adaptive enhancement or attenuation of the influence of specific data points in varying scenarios and data densities, thereby achieving superior filtering outcomes.

Although both the KNN_PCF algorithm and the Hy_WHF algorithm demonstrate strong performance in processing point cloud data from offshore oil and gas platforms, each algorithm possesses specific advantages and limitations. Relying solely on a single filtering algorithm may not fully exploit its potential or yield optimal filtering outcomes in certain situations. To address this, this paper recognizes the complementary nature of these filtering techniques and proposes the exploration of an effective combination between the KNN_PCF algorithm and the Hy_WHF approach. This integration aims to develop a weighted mixed filtering algorithm that can deliver more efficient and accurate filtering results.

In addition to proposing and validating filtering algorithms, this research goes beyond and examines the practical application effects of these algorithms on offshore oil and gas platforms. To achieve this, the Poisson implicit surface reconstruction algorithm [23] is employed for 3D reconstruction of point cloud data obtained from offshore oil and gas platforms [24]. The main objective is to address the challenge of land-based prefabrication for these platforms [25] by providing more reliable dimensional information for the engineering model of “prefabrication on land, installation at sea”. This approach aims to enhance the accuracy and efficiency of construction processes, ultimately contributing to the improved execution of offshore oil and gas platform projects [26].

## 2. Materials and Methods

### 2.1. Data Collection

**Figure 1:**
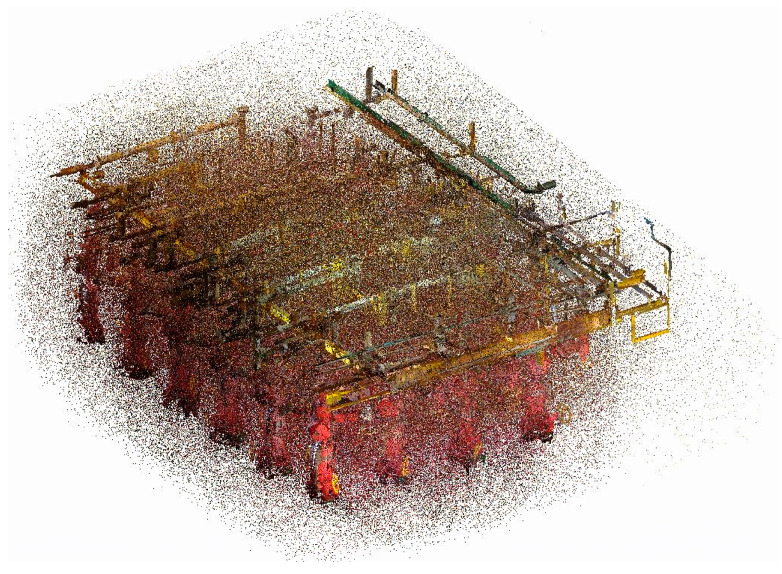
Point cloud data of offshore oil and gas platforms.

**Figure 2:**
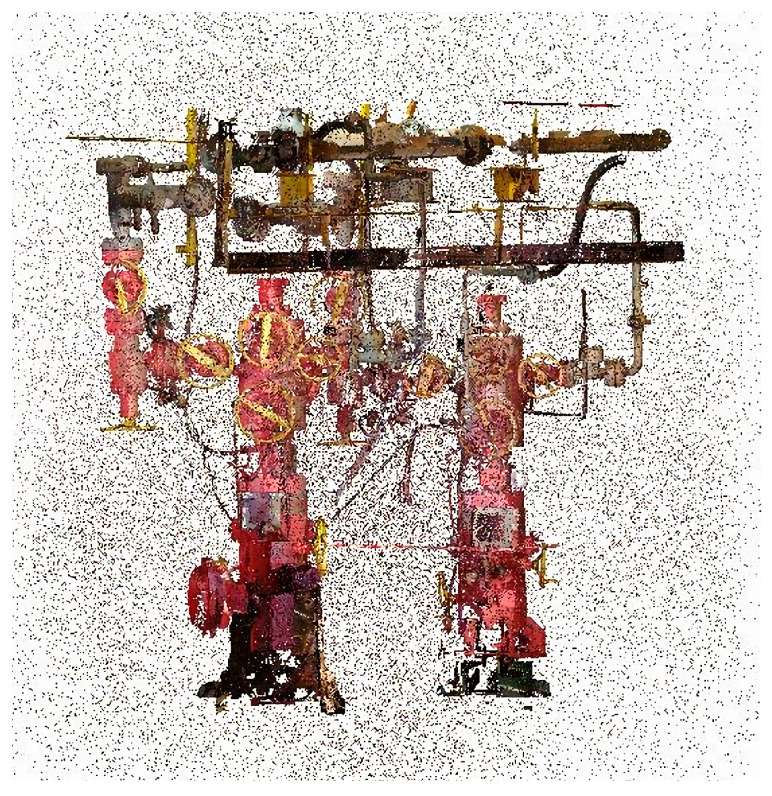
Point cloud data of selected offshore oil and gas platforms.

To address issues such as isolated points, surface noise, and surface reflection noise in the point cloud data sets of offshore oil and gas platforms, this study—on 10 August 2022—used an xFARO s350 model 3D laser scanner (FARO, Lake Mary, FL, USA) to acquire point cloud data from three offshore platforms in the Xi Jiang oil field. The study presents the overall point cloud data of the offshore oil and gas platforms (Figure 1) the partial point cloud data of an offshore oil and gas platform (Figure 2). This approach demonstrates how modern 3D scanning technology can capture intricate details of such large-scale industrial structures, providing a comprehensive dataset for further analysis and application in structural assessment and planning.

### 2.2. Methods

To mitigate challenges associated with isolated points, surface noise, and surface reflection noise in point cloud datasets of marine engineering equipment, this paper proposes the utilization of a combination of filtering algorithms, namely KNN_PCF (K-Nearest-Neighborhood point cloud filtering) and Hy_WHF (hyperbolic weighted hybrid filtering). By implementing this approach, the objective is to achieve a smoother representation of the point cloud data while effectively removing noise points.

#### 2.2.1. K-Nearest-Neighborhood Point Cloud Filtering

**Figure 3:**
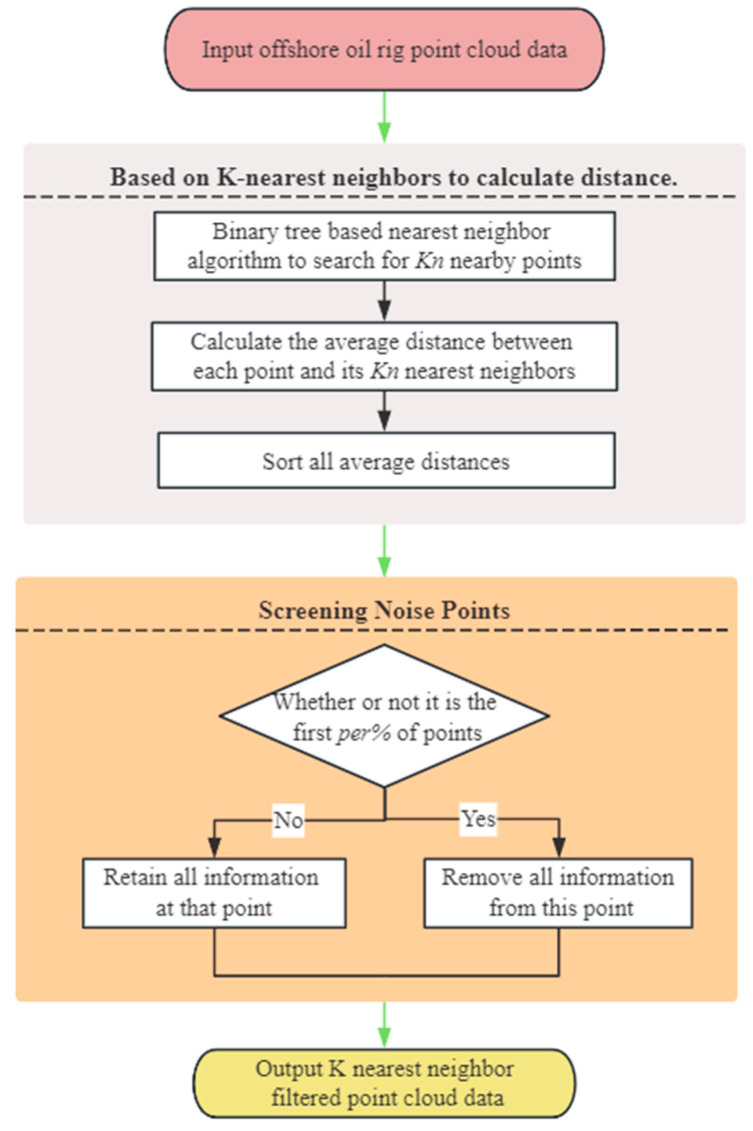
Flowchart of K-Nearest-Neighborhood point-cloud-filtering-based algorithm.

In the KNN_PCF step, point cloud data are processed to remove outliers and isolated points. The flowchart of the KNN_PCF algorithm is shown in Figure 3.

Initially, to efficiently locate the *K_n_* nearest neighbors for each point, a binary tree data structure is constructed. The KD-tree, a binary tree structure, is employed to partition data points in multi-dimensional space. In the context of a three-dimensional point cloud, we set the dimensionality to *n* = 6, taking into account the *x*, *y*, *z* coordinates, as well as the *r*, *g*, *b* color information of each point. For every point in the point cloud, the binary tree is utilized to identify its *K_n_* nearest neighbors. Taking the central point $p_{c,i}$ as an example, its set of *K_n_* nearest neighbors is $\left\{p_{c,i}^{1},p_{c,i}^{2},\cdots ,p_{c,i}^{Kn}\right\}$. The Euclidean distance, $\rho_{c,i}^{o_{j}}$, between $p_{c,i}$ and its nearest neighbors $p_{c,i}^{o_{j}},\;o_{j}=1,2,\cdots ,Kn$ is computed using Equation (1), where $x_{c,i},\;y_{c,i},\;z_{c,i}$ represent the three-dimensional coordinates of Pc and respectively represent the three-dimensional coordinates of $p_{c,i}^{o_{j}}$.

(1) $$\rho_{c,i}^{o_{j}}=\sqrt{{\left(x_{c,i}-x_{c,i}^{o_{j}}\right)}^{2}+{\left(y_{c,i}-y_{c,i}^{o_{j}}\right)}^{2}+{\left(z_{c,i}-z_{c,i}^{o_{j}}\right)}^{2}},$$

After obtaining the *K_n_* nearest neighbors for each point, the average distance to these neighbors is computed, reflecting the compactness of the local structure around the point. Once the average distances for all points are calculated, a location-based sorting algorithm is employed. Specifically, the largest number, *max_num*, among all average distances is identified, and its number of digits, *max_digits*, is recorded. Starting from the least significant digit, the numbers are sorted in descending order based on their value at that digit. This process continues, moving to higher digits, until all numbers are sorted on the *max_digits* digit. Subsequently, the top *per*% of points based on *max_digits* are removed, where per represents the percentage of data to be deleted, with per being in the range (0, 100). Points with larger distances are considered outliers, and their removal aids in reducing the overall noise level in the data.

Within the KNN_PCF algorithm, the parameter *K_n_* denotes the number of nearest neighbors. A larger *K_n_* value results in more accurate average distance calculations but at a higher computational cost. Conversely, a smaller *K_n_* might yield less accurate average distances, making the results more susceptible to noise, but with reduced computational overhead. The parameter per indicates the percentage of points to be removed from the point cloud. A larger per value results in more points being deleted, potentially leading to loss of information in the filtered point cloud. Conversely, a smaller per value retains more points, making the filtered point cloud closer to the original, but with noise points preserved.

#### 2.2.2. Hyperbolic Function Weighted Fusion Point Cloud Filtering

**Figure 4:**
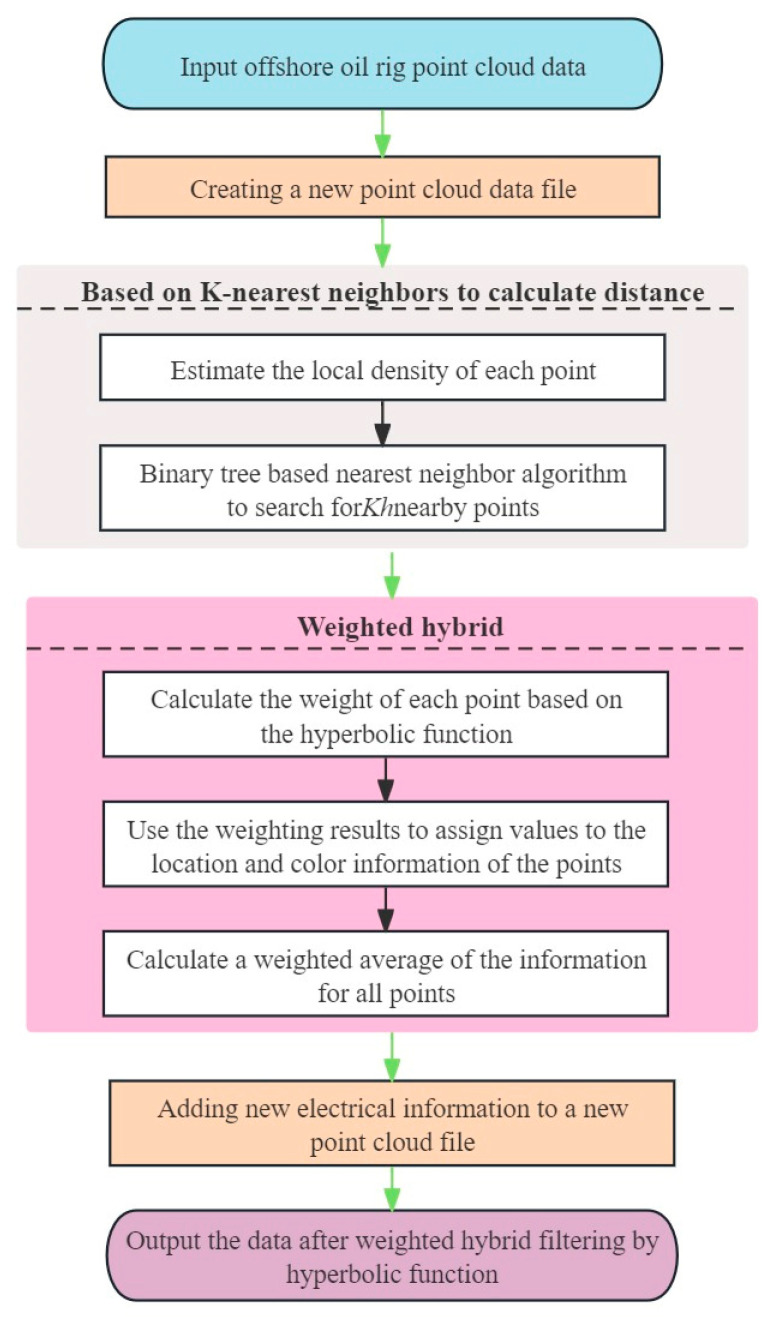
Flowchart of hyperbolic weighted hybrid filtering algorithm.

The hyperbolic function weighted fusion point cloud filtering algorithm aims to eliminate surface noise points in the point cloud and achieve a smoothing effect. The flowchart of this algorithm is depicted in Figure 4.

To perform the weighted mixed filtering, a new point cloud data file is first created to store the point cloud data processed through weighted averaging. During this filtering process, the weight assigned to each point influences its contribution in the weighted average computation. To determine the weight for each point, its local density is estimated. Specifically, the average distance of each point to its nearest neighbors is calculated to gauge the density of points surrounding it. Using a binary tree-based nearest neighbor search algorithm, the indices and squared distances, *d_i*^2^, of the *Kh* nearest neighbors around each point are identified, where *d_i* represents the distance between the *i*th point and its *K_h_* nearest neighbors. The value of *K_h_*, representing the number of neighboring points considered for each point, can be adjusted based on the specific requirements of the application.

To compute the weight factor for each point, a hyperbolic function is defined. This function interpolates between two limit values within its domain. The weight factor, *d_w*, for points in the point cloud is calculated using Equation (2), accepting a parameter *φ*, where *φ* and *d_w*’s value ranges are *φ* ∈ [0,1] and *d_w* ∈ [0,1], respectively. Here, *d_i* represents the average distance between a point and its nearest neighbors, while *φ* is a parameter of the hyperbolic function, adjusting the distribution of the weight factor. The value of *φ*, ranging between 0 and 1, can be adjusted based on the characteristics of the denoised data.

(2) $$\mathrm{d\_w}=\frac{1}{1+\frac{d\_i^{2}}{\phi^{2}}},$$

Once the weight factors for the points are obtained, they are applied to the position and color information of the points to compute the new position and color values. Specifically, a weighted average of the position and color information of neighboring points is taken to derive the new position and color values for each point. This weighted averaging process aids in eliminating surface noise points and achieving smoother data representation.

Finally, the new points obtained through weighted averaging are added to the previously filtered point cloud data, replacing the points in the original point cloud. The result is a point cloud data set that has undergone weighted fusion filtering, containing smoothed point information.

The hyperbolic function-based weighted fusion filtering algorithm, by introducing weight factors and weighted averaging, can reduce surface noise points in point cloud data while maintaining data smoothness. The core idea of this algorithm is to estimate point weights based on local density and adjust the weight factor distribution using a hyperbolic function, achieving effective data processing. Through this algorithm, point cloud data is smoothed and denoised while retaining local features.

In the hyperbolic function-based weighted fusion filtering algorithm, a larger *K_h_* value means more neighboring points are considered, resulting in a smoother outcome but at a higher computational cost. A smaller *K_h_* value retains more details, preserving more noise points. The parameter *φ*, which controls the rate of weight decline, when larger, means weights decline more slowly, and distant points have a more significant influence, leading to a smoother result. A smaller *φ* value means weights decline faster, and only points near the center have a significant influence, leading to a sharper result but retaining more noise points.

#### 2.2.3. Combined Filtering

**Figure 5:**
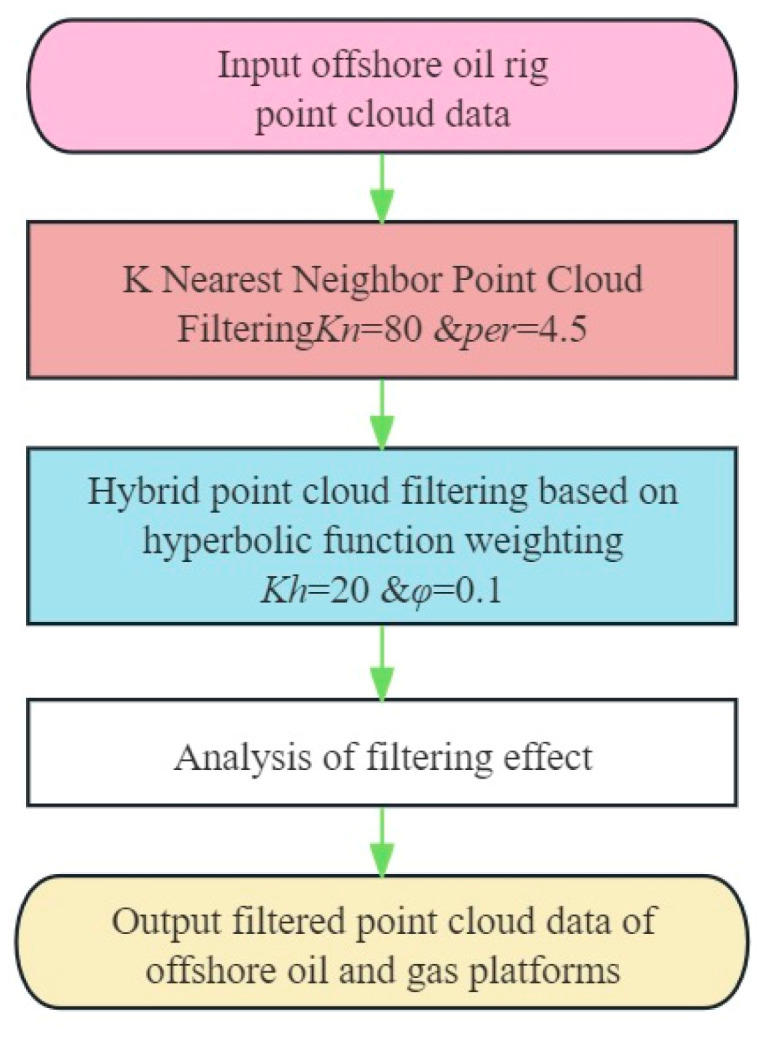
Technical route to a combinatorial filtering algorithm based on a hybrid of K-Nearest-Neighborhood and hyperbolic weighting.

To leverage the strengths of both the KNN_PCF algorithm and the Hy_WHF algorithm, this paper proposes a combined filtering approach. This method first employs the KNN_PCF algorithm for preliminary processing, eliminating points with low local density or isolated points. Subsequently, the data filtered through KNN_PCF are passed to the Hy_WHF algorithm, further smoothing the data while retaining features with geometric and topological continuity. The combined filtering technique’s flowchart is illustrated in Figure 5.

This combined filtering approach capitalizes on the strengths of both filtering algorithms, compensating for the limitations of each. While the KNN_PCF algorithm effectively removes outliers and isolated points, it might lead to excessive smoothing in certain areas. On the other hand, the Hy_WHF algorithm can smooth data while retaining details but might not perform well with larger noise points. By integrating both algorithms, the point cloud data retains its geometric shape and local features while eliminating most noise points, resulting in cleaner, smoother, and higher fidelity point cloud data. 

## 3. Experiments

### 3.1. Experimental Environment

All experiments in this paper were conducted on workstations with uniform standard configurations. The hardware and software configurations used for the experiments are detailed in Table 1. These configurations are common industry standards and not specially customized or experimental in nature, ensuring the generality and reproducibility of the experimental results. 

### 3.2. Experimental Evaluation Parameters

This study introduces several key performance indicators, such as the target point retention rate [27], noise point screening rate, denoising rate, and total error rate [28]. These indicators are utilized to assess the important performance of filtering algorithms in effectively removing noise points and preserving target points. Additionally, a consistency assessment metric [29] is employed to evaluate the similarity between the point cloud data and the three-dimensional reconstruction model.

In the point cloud filtering analysis, the original point cloud (denoted as O) is divided into target point cloud (O*_t_*) and noise point cloud (O*_n_*). The point cloud obtained after filtering (denoted as R) is also divided into two subsets: retained target point cloud (R*_t_*) and residual noise point cloud (R*_n_*). Meanwhile, the points removed during the filtering process (denoted as D) are divided into mistakenly removed target point cloud (D*_t_*) and successfully removed noise point cloud (D*_n_*).

(1) Target Point Retention Rate (TR): The target point retention rate is an indicator measuring the completeness of the retained target point cloud. A higher TR indicates that the filtering algorithm performs better in retaining essential information. Its formula is:

(3) $$TR=\frac{R_{t}}{D_{t}+R_{t}+R_{n}}\times 100\%,$$

(2) Noise Point Screening Rate (NSR): The noise point screening rate measures the efficiency of noise point removal. A higher NSR indicates that the filtering algorithm performs better in noise removal, retaining more useful information. Its formula is:

(4) $$NSR=\frac{D_{n}}{D_{n}+D_{t}+R_{n}}\times 100\%,$$

(3) Denoising Rate (R*_d_*): The denoising rate indicates the proportion of noise points successfully removed by the filtering algorithm. A higher R*_d_* suggests better algorithm performance. Its formula is:

(5) $$R_{d}=\frac{D_{n}}{O_{n}}\times 100\%,$$

(4) Total Error (E*_t_*): The total error indicates the combined error from the filtering algorithm mistakenly removing target points as noise and retaining noise points as target points. A smaller E*_t_* indicates better algorithm performance. Its formula is:

(6) $$E_{t}=\frac{D_{t}+R_{n}}{O}\times 100\%,$$

These evaluation metrics can be employed to assess the noise removal effectiveness of filtering algorithms and the degree to which the morphology of the offshore equipment point cloud is maintained, facilitating the selection of the most suitable filtering algorithm and parameters for specific application scenarios.

In the analysis of point cloud three-dimensional reconstruction, consistency assessment focuses on measuring the distance between the point cloud and the surface of the reconstructed model. The process involves obtaining the three-dimensional reconstruction model of the input point cloud and calculating the distance from each point in the point cloud to the surface of the reconstructed model. This is typically achieved by calculating the Euclidean distance [30] to the nearest point (or triangle) on the surface. Finally, the mean distance and the standard deviation [31] of all points to the surface are computed. The mean distance provides an indication of the overall consistency between the point cloud and the reconstruction model, while the standard deviation reflects the variability in the distribution of distances. Generally, lower mean distances and standard deviations signify a higher level of consistency.

### 3.3. Experimental Process and Results

The experimental method of this study is systematically structured into three main parts to evaluate and compare the effectiveness of various filtering algorithms in processing point cloud data for offshore oil and gas platforms.

In the first part, two conventional filtering algorithms, the statistical filtering algorithm and the radius filtering algorithm, were applied to a section of the point cloud data from the same offshore oil and gas platform. In addition, this study introduced two novel algorithms: the KNN_PCF (K-Nearest-Neighborhood point cloud filtering) algorithm and the Hy_WHF (hyperbolic weighted hybrid filtering) algorithm. To optimize their performance, we employed a controlled variable method for parameter tuning. Each algorithm’s parameters were systematically adjusted, and multiple comparative experiments were conducted. The primary objective of this phase was to identify the most effective parameter settings for each algorithm, especially to assess the KNN_PCF algorithm’s suitability for this specific dataset.

In the second part, building on the findings from the previous experiments, we established the optimal parameter settings for each algorithm when applied to offshore oil and gas platform data. This phase involved experimenting with both individual and combined applications of the KNN_PCF and Hy_WHF algorithms as well as exploring the synergy between the radius and statistical filtering algorithms. The focus was to validate the efficiency of the combined filtering approach, particularly comparing the noise reduction capabilities of the KNN_PCF and Hy_WHF method against the radius–statistical filtering method.

Finally, the third part of the study utilized the Poisson algorithm to perform 3D reconstruction of the offshore oil and gas platform datasets, pre- and post-denoising. The reconstructions were meticulously compared to evaluate the impact of the various filtering algorithms on the final 3D model quality. This comparative analysis aimed to provide insights into the efficacy of the filtering techniques in enhancing the accuracy and detail of the 3D models of offshore platforms.

#### 3.3.1. Individual Filtering Algorithms

To precisely determine the optimal parameter settings for the offshore oil and gas platform point cloud dataset under different algorithms, this study conducted detailed comparative experiments on statistical filtering, radius filtering, the KNN_PCF algorithm, and the Hy_WHF algorithm, using the part of the offshore oil and gas platform point cloud data shown in Figure 2. The experiments with these four algorithms were compared horizontally, verifying the efficiency of the KNN_PCF algorithm in removing noise from the point cloud data of offshore oil and gas platforms.

1. Algorithm Parameterization

(1) Statistical Filtering

Initially, the standard deviation multiple *σ* is fixed at 1.5, and the range for the number of neighborhood search points *K_s_* is set between 6 and 10, with a step size of 2. This allows for a quantitative estimation of the denoising effect under these parameter settings. Through this process, the optimal value for *K_s_* can be determined. Subsequently, to further optimize the parameter settings, the range for *σ* is set between 1.0 and 2.0—with a step size of 0.5—to ultimately ascertain the best value for *σ*. Using this method, we can employ various parameter combinations to achieve the best performance of statistical filtering on the offshore oil and gas platform point cloud data. For each parameter combination of statistical filtering, its precision evaluation parameter values are calculated (Table 2) (Note: In all tables, bolded rows represent the best algorithm parameters and their corresponding evaluation values, following the same convention).

The table shows the evaluated parameter values of statistical filtering algorithms with different parameter combinations under the point cloud data of offshore oil and gas platforms, and it can be seen from Table 2 that the denoising of point cloud data is best when the number of neighborhood search points *K_s_* is 8 and the standard deviation multiplier *σ* is 1.5.

(2) Radius Filtering

In the analysis of radius filtering, we first fixed the radius *r* to be 0.08 and set the range of the threshold of the number of neighboring points *K_r_* to be between 60 and 120 with a step size of 30 as a way to quantitatively estimate the denoising effect under different parameters and to determine the optimal value of *K_r_*. Subsequently, in order to further optimize the parameters, we adjusted the value of *r* to a range of 0.06 to 0.10 with a step size of 0.02 so as to finally determine the optimal value of *r*. In this way, we are able to use individual parameter combinations to obtain the best performance of radius filtering on offshore oil and gas platform point cloud data. For each parameter combination result of the radius filtering, its accuracy evaluation parameter value was calculated (Table 3).

The table shows in detail the values of various performance parameters of the radius filtering algorithm under different parameter combinations. As can be seen from Table 3, when *K_r_* is 90 and *r* is 0.08, the denoising of point cloud data is the best, its target point retention rate is 99.069%, the noise point filtering rate is 82.357%, the denoising rate is 86.082%, and the total error rate is 0.892%.

(3) K-Nearest-Neighborhood Point Cloud Filtering (KNN_PCF)

During the experiment of denoising offshore oil and gas platform point cloud data with the KNN_PCF algorithm, the percentage (*per*) was initially set at 4.5, and the range for the number of nearest neighbors (*K_n_*) was set between 70 and 90, with a step size of 10. Through these settings, we could quantitatively estimate the denoising effects under different parameters and thus determine the optimal value for *K_n_*. Subsequently, we adjusted the range of *per* to be between 4.0 and 5.0, with a step size of 1.0, to find the most suitable value for *per*. Using this method, we could employ various parameter combinations to achieve the best performance of the KNN_PCF algorithm on the offshore oil and gas platform point cloud data. For each combination of parameters, we calculated its precision evaluation parameter values (Table 4).

The table shows the evaluation parameter values of the KNN_PCF algorithm with different parameter combinations for point cloud data of offshore oil and gas platforms. From Table 4, it can be seen that when the number of nearest neighbors (*K_n_*) is 80 and the fixed percentage (*per*) is set at 4.5, the denoising effect on the point cloud data is the best.

(4) Hyperbolic Weighted Hybrid Filtering (Hy_WHF)

During the Hy_WHF (hyperbolic weighted hybrid filtering) algorithm experiment, we initially fixed the parameter *φ* at 0.1 and set the range for the number of nearest neighbors (*K_h_)* between 15 and 25, with a step size of 5. This was done to quantitatively estimate the denoising effects under different parameters and to determine the best value for *K_h_*. Then, we adjusted the range of *φ* to be between 0.08 and 0.12, with a step size of 0.02, to ultimately find the most suitable value for *φ*. Through this process, we could use various parameter combinations to achieve the best performance of the Hy_WHF algorithm on the point cloud data of offshore oil and gas platforms. For each combination of parameters, we calculated its precision evaluation parameter values (Table 5).

The table provides detailed performance parameter values for the Hy_WHF algorithm under different parameter settings. From Table 5, it is evident that when the number of nearest neighbors (*K_h_*) is 20 and the parameter *φ* is set at 0.1, the denoising effect on the offshore oil and gas platform point cloud data is the best.

2. Comparison of Different Algorithms

**Figure 6:**
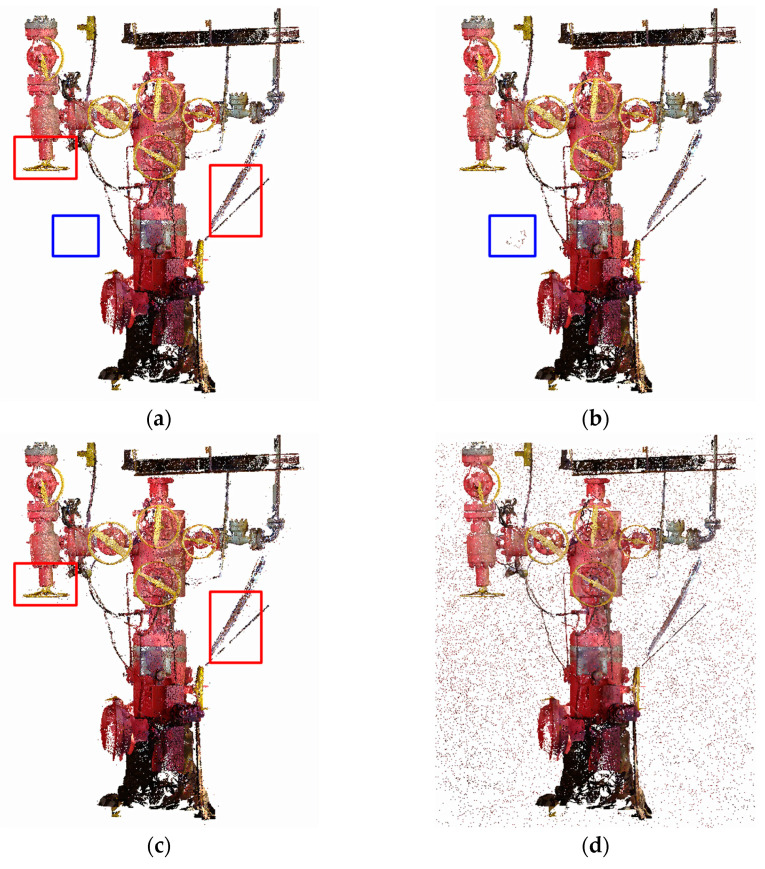
Visualization results based on different filtering algorithms. (**a**) KNN_PCF algorithm filtered point cloud data; (**b**) statistical filtered point cloud data; (**c**) radius-filtered point cloud data; (**d**) Hy_WHF algorithm filtered point cloud data.

To more thoroughly validate the effectiveness of the KNN_PCF algorithm in removing noise from point cloud data of offshore oil and gas platforms, this study employed a precise experimental method. We used the optimal parameters for each algorithm as determined by meticulous experiments earlier in the paper as a benchmark. Specifically, we selected statistical filtering with parameters *K_s_* = 8, *σ* = 1.5; radius filtering with parameters *K_r_* = 90, *r* = 0.08; KNN_PCF algorithm with parameters *K_n_* = 80, *per* = 4.5; and Hy_WHF algorithm with parameters *K_h_* = 20, *φ* = 0.1. We conducted a detailed comparison of their filtering effects using various indicators and visual analysis. A table of filtering evaluation parameters based on different algorithms was also provided (Table 6). To visually demonstrate the denoising effects of different filtering algorithms on point cloud data, the denoising results were visualized (Figure 6).

From the evaluation parameter Table 6 in the comparative experiments, it is apparent that the KNN_PCF algorithm excels in terms of target point retention rate (TR), noise point screening rate (NSR), and denoising rate (R***_d_***), achieving 99.485%, 89.987%, and 91.417%, respectively. It has the smallest total error of only 0.492. Moreover, the noise point screening rate obtained using the KNN_PCF algorithm is about 7 percentage points higher than that of the radius filtering algorithm, with a total error result that is 0.4 percentage points lower. However, when using the Hy_WHF algorithm alone, its denoising effect is comparatively poorer.

Figure 6 illustrates the selection of regions using blue boxes to highlight the presence of noise points that are not effectively eliminated through statistical filtering but can be successfully removed using the KNN_PCF algorithm. Moreover, red boxes are employed to identify areas where noise persists despite the application of radius filtering but can be effectively addressed by utilizing the KNN_PCF algorithm. By comparing the visualization results of different filtering algorithms in Figure 6, we can see that statistical filtering effectively removes most noise points, but its handling of dense clustered noise and surface noise is less satisfactory. Radius filtering improves the processing of densely clustered noise points, but also lacks in preserving surface detail of the data. The KNN_PCF algorithm combines the advantages of radius filtering and statistical filtering, effectively removing both dense clustered noise and surface noise.

#### 3.3.2. Combined Filtering Algorithms

Based on the outcomes of previous experiments, it was observed that the KNN_PCF algorithm demonstrated superior performance in comparison to radius and statistical filtering methods in processing point cloud data from offshore oil and gas platforms. However, while the Hy_WHF algorithm individually showed less favorable results than the other algorithms, its combination with the KNN_PCF algorithm was explored to potentially enhance overall filtering effectiveness. Additionally, experiments were conducted to combine radius and statistical filtering methods, aiming to investigate their synergistic effects in different scenarios. This approach also served to provide a comparative benchmark for the combined use of the KNN_PCF and Hy_WHF algorithms.

In this experiment, we used the optimal parameters of each algorithm, as determined through meticulous previous experiments, as a benchmark. We further validated the combined filtering method based on KNN_PCF and Hy_WHF (selecting parameters *K_n_* = 80, *per* = 4.5, and *K_h_* = 20, *φ* = 0.1) for its effectiveness in filtering point cloud data of offshore oil and gas platforms. Additionally, we compared this method to the combined filtering method based on radius and statistics (selecting parameters *K_s_* = 8, *σ* = 1.5, and *K_r_* = 90, *r* = 0.08) in terms of denoising effects.

**Figure 7:**
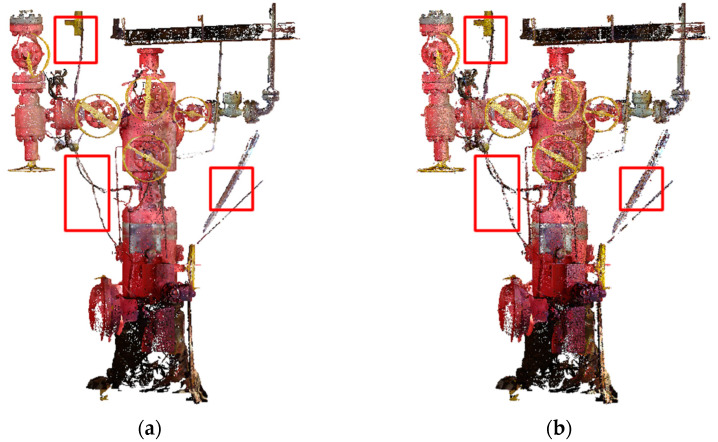
Visualization results based on different combined filtering algorithms. (**a**) Combined filtering algorithm based on the KNN_PCF and Hy_WHF; (**b**) combined based on the statistics and radius filtering.

**Figure 8:**
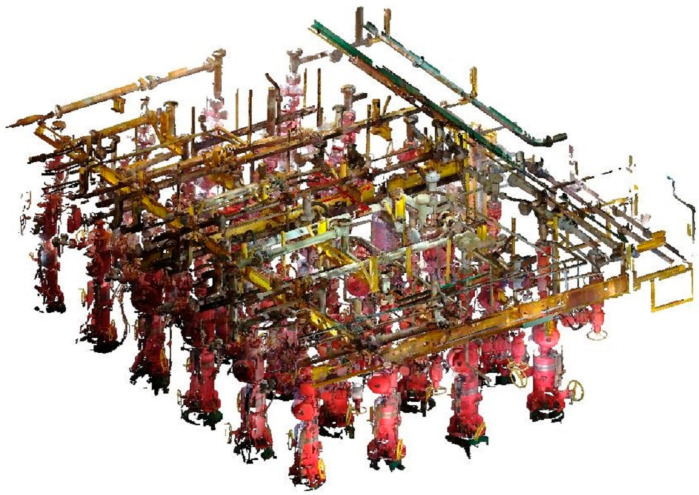
Offshore oil rig point cloud data after combined filtering algorithm based on KNN_PCF and Hy_WHF.

To clearly demonstrate the differences between different combined filtering algorithms, we conducted a detailed comparison of their filtering effects using various indicators and visual analysis. A table of filtering evaluation parameters based on different combined algorithms (Table 7) was listed. The denoising effects of different filtering algorithms on point cloud data were visually demonstrated, with areas of noise points not removed after filtering highlighted in red rectangular frames (Figure 7). Additionally, a visualization of the offshore oil and gas platform point cloud data after combined filtering based on the KNN_PCF and Hy_WHF algorithms was shown (Figure 8). 

From Table 7, it is evident that the combined filtering of point cloud data based on the KNN_PCF and Hy_WHF algorithms performs better than the combined filtering algorithm based on statistics and radius in three evaluation parameters: target point retention rate (TR), noise point screening rate (NSR), and denoising rate (R***_d_***). Specifically, in terms of noise point screening rate (NSR), the combined filtering effect of the KNN_PCF and Hy_WHF algorithms is significantly superior to the method based on statistics and radius, with the former being about 11% higher. In terms of the error rate (E*_t_*) evaluation parameter, the performance of the combined filtering based on KNN_PCF and Hy_WHF algorithms (0.206%) far exceeds that of the combined method based on statistics and radius (0.781%).

The regions selected using the red boxes indicate noise points that cannot be rejected by the combined radius- and statistical-filtering-based algorithm but can be rejected by the combined KNN_PCF- and Hy_WHF-based algorithm, and the smoothing effect of the combined KNN_PCF- and Hy_WHF-based algorithm is clearly visible in these regions. The visualization results in Figure 7 clearly demonstrate the superiority of the combined filtering algorithm based on KNN_PCF and Hy_WHF over traditional combined methods. The significant reduction of noise points in the areas highlighted by red rectangular frames and the better preservation of details are evident.

Figure 8 shows the denoising effect of the combined filtering algorithm based on KNN_PCF and Hy_WHF on the entire offshore oil and gas platform dataset. We can see that the combined algorithm of this study can almost remove all dense clustered noise, surface noise points, and discrete points as well as eliminate light reflection noise, resulting in more accurate color information in the point cloud data.

#### 3.3.3. Three-Dimensional Reconstruction of Point Cloud Data

First, we extracted the point cloud data of pipes and flanges from both the original point cloud data and the data processed by the optimal combined filtering algorithm and then used the Poisson reconstruction algorithm for 3D reconstruction [32]. Since the actual scanned data of the offshore oil and gas platform did not have corresponding real model data of the components, we selected visual analysis and consistency assessment as two indicators to validate the modeling effects.

**Figure 9:**
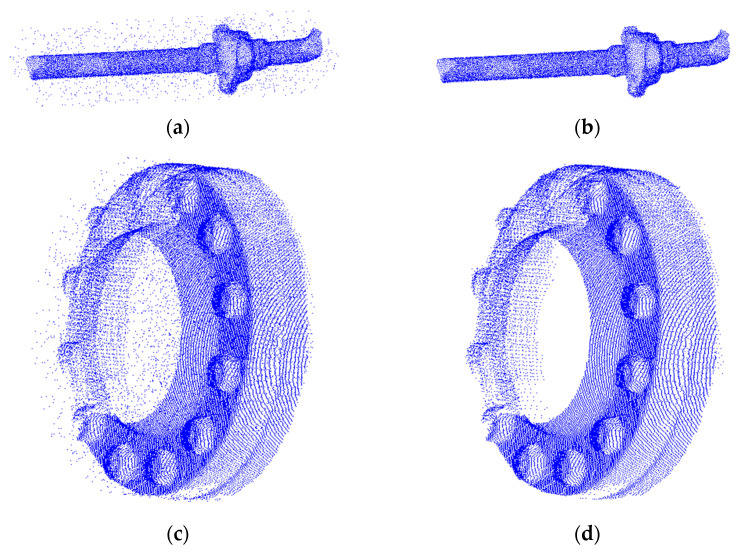
Marine engineering equipment components original point cloud. (**a**) Pipeline original point cloud; (**b**) pipeline post-filter point cloud; (**c**) flange original point cloud; (**d**) flange post-filter point cloud.

**Figure 10:**
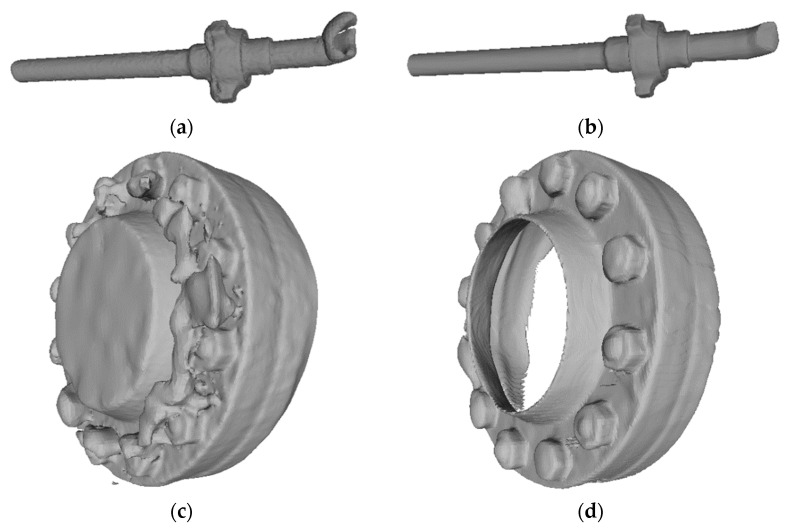
Three-dimensional reconstruction results of offshore engineering equipment components. (**a**) Pipeline original point cloud reconstruction results; (**b**) pipeline filtered point cloud reconstruction results; (**c**) flange original point cloud reconstruction results; (**d**) flange filtered point cloud reconstruction results.

Using pipes and flanges as examples, we performed 3D reconstructions of the data before and after filtering. The results of the 3D reconstruction precision evaluation parameters for different point cloud data sources are shown in Table 8. We visually presented the point cloud images of marine equipment components (Figure 9) and the reconstruction results of marine equipment component point cloud data (Figure 10).

From Table 8, it can be seen that the consistency of the results reconstructed from the filtered point cloud data is relatively high. After processing with the filtering algorithms of this study, the average distance and standard deviation for the pipe decreased by 3.28 mm and 0.047, respectively, while for the flange, they reduced from 8.26 mm to 1.85 mm, with the standard deviation decreasing to 0.002.

As can be seen in Figure 9, the point cloud data before denoising with the filtering algorithm have more noise points. This has a greater impact on the post-processing of the point cloud data.

From Figure 10, it is evident that the models reconstructed from unfiltered point cloud data have rough and uneven surfaces with many protrusions and depressions, especially at the edges and in detailed parts of the model. In contrast, the 3D reconstruction results after processing with the filtering algorithms of this study show a much smoother and finer surface as well as clear structural edges.

## 4. Discussion

The previous sections conducted experiments on point cloud data obtained from offshore oil and gas platforms. Individual filtering algorithms were employed to process the data, validating the suitability of the KNN_PCF algorithms for this specific dataset. Additionally, point cloud data filtering techniques were combined to enhance the quality of the dataset, and 3D surface reconstruction experiments were conducted on point cloud data captured from equipment components. In the following section, we will present and analyze the results obtained from these experiments.

### 4.1. Discussion on Individual Filtering Algorithm Experiments

In these experiments, we initially evaluated various filtering algorithms on offshore oil and gas platforms to identify the optimal parameter settings for each algorithm. Subsequently, we assessed the effectiveness of KNN_PCF algorithms in noise removal from offshore oil and gas platform point cloud data. In the following section, we will discuss the individual experiments in detail.

(1) When selecting the optimal parameters for each algorithm, it becomes crucial to ensure the choice of neighboring points that are relatively appropriate, accurately assess the density and noise levels of the local area, minimize misjudgments, and maintain structural integrity to avoid losing important surface details during the denoising process [33].
(2) This experiment aimed to compare the effectiveness of four distinct point cloud filtering methods in denoising offshore oil and gas platform data. Based on the obtained results (Table 5), the KNN_PCF algorithm demonstrated superior performance compared to the other algorithms. It exhibited higher rates of target point retention, noise point screening, and denoising, leading to the lowest total error rate. The superiority of the KNN_PCF method can be attributed to its ability to leverage local structural information [34]. By considering the density and distribution of neighboring points, this method can more accurately differentiate between noise points and target points.
(3) Statistical filtering performs well in noise removal but is less effective in handling dense clustered noise and surface noise [35]. This is due to the fact that statistical filtering is mainly based on the global statistical information of the point cloud and cannot distinguish local dense noise in detail [36]. Global statistical filtering ignores local details [33].
(4) Radius filtering has demonstrated improvements in handling densely clustered noise; however, it falls short in preserving data surface details. The underlying reason for this limitation is that radius filtering utilizes the count of points within a fixed radius to identify noise. Consequently, when dealing with point clouds of uneven density, radius filtering could mistakenly remove fine surface details [19].
(5) When utilized independently, the Hy_WHF algorithms demonstrate unsatisfactory performance in scenarios with a high volume of noise points. This limitation primarily stems from the algorithm’s weighted hybrid principle, which is easily disrupted in environments with dense noise. However, the Hy_WHF algorithm is particularly adept in contexts in which noise points are relatively fewer and closer to the target point cloud. In such scenarios, its approach to balancing noise removal with the preservation of target points proves to be more effective. This is because the Hy_WHF algorithm is designed to handle surface noise points adeptly, excelling in environments where noise points are not overwhelmingly dense but are proximal to the target data [37].

### 4.2. Discussion on Combined Filtering Algorithm Experiments

This study revealed that the integrated approach, combining both radius filtering and statistical filtering algorithms, outperforms the usage of individual algorithms such as radius filtering or statistical filtering alone when processing point cloud data in large and complex scenes. The strength of this combined method lies in its ability to leverage the spatial consistency offered by radius filtering and the data distribution characteristics provided by statistical filtering. Consequently, this integrated approach facilitates the efficient identification of complex structures and effective removal of noise [38].

The experimental results additionally demonstrate that the Hy_WHF algorithm exhibits noteworthy effectiveness in environments with a minimal number of noise points. It particularly excels in eliminating noise points that are adjacent to the target point cloud, yielding noticeable smoothing effects. Moreover, it presents distinct advantages in handling noise points originating from light reflection and successfully restores color information in point clouds [39]. Consequently, combining this algorithm with the KNN_PCF algorithm not only safeguards the detailed information of the point cloud but also substantially enhances the efficiency of denoising operations.

Further comparative experiments have revealed that the KNN_PCF algorithm surpasses traditional radius and statistical filtering algorithms in terms of retention rate and noise screening efficiency. Therefore, combining the KNN_PCF with the Hy_WHF algorithm forms a combined approach that exhibits exceptional performance in processing point cloud data of offshore oil and gas platforms. This combined method proves to be more efficient in recognizing complex structures and handling noise compared to the simple combination of radius and statistical filtering algorithms. The combined approach showcases superior denoising results and preservation of structural details.

### 4.3. Discussion on 3D Reconstruction of Point Cloud Data Experiment

The experimental results clearly demonstrate that the optimal combined filtering algorithm used in this study played a key role in enhancing the accuracy of the 3D reconstructed models. This enhancement is evident not only in the reduction of average distance error and standard deviation but also, more importantly, in the significant improvement in the consistency of the reconstruction results [40]. The improvement is underpinned by the ability of the filtering algorithm to effectively remove noise while preserving key structural details of the model [41]. This is particularly crucial in the reconstruction of complex components such as pipes and flanges, where preserving details and removing noise is essential. As a result, the 3D models exhibit not only smoother and finer surfaces but also clearer edges, more accurately reflecting the actual shape and size of objects during the reconstruction process.

Further analysis reveals that the meticulous execution of the filtering algorithm in handling model edges and details effectively mitigates surface roughness and geometric distortion caused by noise points. This is particularly evident in the comparison of models reconstructed from unfiltered point cloud data with those processed in Figure 10. Models reconstructed from unfiltered data exhibit rough and uneven surfaces, with many protrusions and depressions at the edges and in detailed parts. In contrast, models processed through the filtering algorithm show much smoother surfaces and clear structural edges. This contrast highlights the importance of the filtering algorithm in 3D reconstruction, especially its ability to accurately reconstruct details and remove noise in large-scale point cloud data.

## 5. Conclusions

This study successfully implemented several point cloud filtering algorithms for offshore oil and gas platforms and rigorously evaluated their performance through a series of experiments. The following conclusions were drawn:

(1) The study conducted a comprehensive evaluation of commonly used statistical and radius filtering algorithms, as well as the KNN_PCF and Hy_WHF algorithms proposed in this paper. The results indicate that while traditional filtering algorithms are generally effective, they have limitations in handling complex structures present in point cloud data from offshore oil and gas platforms. Specifically, traditional filtering approaches struggle to effectively address noise in densely populated areas of complex scenes, maintain the preservation of fine structural details, and adapt to the heterogeneity of point cloud data;
(2) The combined filtering algorithm, which integrates KNN_PCF and Hy_WHF, offers substantial advantages compared to other single or combined filtering methods. It demonstrates superior performance in noise removal, enhancing smoothness, preserving the fidelity of point cloud data, addressing light reflection noise, and restoring the original colors of objects;
(3) In denoising point cloud data of complex scenes, the effectiveness of combined filtering is more pronounced compared to single filtering methods;
(4) Point cloud data processed with the combined filtering method can generate high-quality 3D models, showing advantages in complex 3D reconstruction of offshore oil and gas platforms.

While the current research has made notable progress, challenges in processing point cloud data for large scenes such as offshore oil and gas platforms, which are prone to various types of noise, still remain unresolved. Further research and exploration are therefore necessary. In order to enhance the accuracy and stability of filtering algorithms, future studies could explore the combination of deep learning techniques and other advanced computational methods. This approach would allow for the extraction of deeper features and patterns from the data, ultimately leading to improved algorithm performance.

## Figures and Tables

**Table 1:** Software and hardware configuration.

Name	Configuration
Operating System	Ubuntu 18.04
Processor	Intel@Xeon(R)W-2235 CPU@3.80 GHz x 12
Graphics Card	NVIDIA Quadro RTX4000 (Santa Clara, CA, USA)
Video Memory	8 GB
Development Environment	Anaconda 3
Compilation Environment	PyCharm 2022.2.1
Python Version	3.7.3
PCL Version	1.9.1
VTK Version	8.2.0
QtCreator Version	5.9.7

**Table 2:** Evaluation for different parameters of statistical filtering algorithm.

Algorithm Parameterization	TR (%)	NSR (%)	R*_d_* (%)	E*_t_* (%)
***K_s_* = 8 and *σ* = 1.5**	**98.552**	**71.344**	**71.892**	**1.397**
*K*_s_ = 6 and *σ* = 1.5	98.410	68.859	70.188	1.536
*K_s_* = 10 and *σ* = 1.5	98.329	67.109	68.120	1.615
*K_s_* = 8 and *σ* = 1.0	98.422	68.965	69.993	1.524
*K_s_* = 8 and *σ* = 2.0	98.285	66.067	66.759	1.659

**Table 3:** Evaluation for different parameters of the radius filtering algorithm.

Algorithm Parameterization	TR (%)	NSR (%)	R*_d_* (%)	E*_t_* (%)
***K_r_* = 90 and *r* = 0.08**	**99.069**	**82.357**	**86.082**	**0.892**
*K_r_* = 60 and *r* = 0.08	98.783	76.876	80.325	1.169
*K_r_* = 120 and *r* = 0.08	98.248	71.928	88.785	1.676
*K_r_* = 90 and *r* = 0.06	98.261	71.084	84.690	1.668
*K_r_* = 90 and *r* = 0.1	98.709	76.465	83.155	1.238

**Table 4:** Evaluation based on different parameters of KNN_PCF algorithm.

Algorithm Parameterization	TR (%)	NSR (%)	R*_d_* (%)	E*_t_* (%)
***K_n_* = 80 and *per* = 4.5**	**99.485**	**89.987**	**91.417**	**0.492**
*K_n_* = 90 and *per* = 4.5	99.458	89.481	91.144	0.518
*K_n_* = 70 and *per* = 4.5	99.443	89.150	90.337	0.532
*K_n_* = 80 and *per* = 4.0	99.118	82.522	82.588	0.846
*K_n_* = 80 and *per* = 5.0	98.842	87.521	90.043	1.317

**Table 5:** Evaluation for different parameters of the Hy_WHF algorithm.

Algorithm Parameterization	TR (%)	NSR (%)	R*_d_* (%)	E*_t_* (%)
***K_h_* = 20 and *φ* = 0.1**	**98.673**	**73.607**	**73.749**	**1.279**
*K_h_* = 15 and *φ* = 0.1	98.510	70.264	70.265	1.439
*K_h_* = 25 and *φ* = 0.1	97.607	71.222	72.228	1.344
*K_h_* = 20 and *φ* = 0.08	98.382	67.680	67.712	1.565
*K_h_* = 20 and *φ* = 0.12	98.6131	72.392	72.522	1.438

**Table 6:** Filtering evaluation parameters for different algorithms.

Algorithm Parameterization	TR (%)	NSR (%)	R_d_ (%)	E_t_ (%)
**KNN_PCF**	**99.485**	**89.987**	**91.417**	**0.492**
Hy_WHF	98.673	73.607	73.749	1.279
Statistical filtering	98.552	71.344	71.892	1.397
Radius filtering	99.069	82.357	86.082	0.892

**Table 7:** Filtering evaluation parameters based on different combined algorithms.

Algorithm	TR (%)	NSR (%)	R*_d_* (%)	E*_t_* (%)
**Combined Filtering Based on** **the KNN_PCF and Hy_WHF Algorithms**	**99.784**	**95.736**	**95.742**	**0.206**
Combined Filtering Based on the Radius and Statistical Filtering	99.183	84.584	88.613	0.781

**Table 8:** Accuracy evaluation of 3D reconstruction.

Part Type	Source of Point Cloud Data	Mean Distance/mm	Standard Deviation
Pipeline	Original Point Cloud	7.30	0.078
	**Post-Filter Point Cloud**	**4.02**	**0.031**
Flange	Original Point Cloud	8.26	0.091
	**Post-Filter Point Cloud**	**1.85**	**0.002**

## Data Availability

The data presented in this study are available on request from the corresponding author.
